# Perinatal Depression in Australian Women during the COVID-19 Pandemic: The Birth in the Time of COVID-19 (BITTOC) Study

**DOI:** 10.3390/ijerph19095062

**Published:** 2022-04-21

**Authors:** Belinda Lequertier, Mia A. McLean, Sue Kildea, Suzanne King, Hazel Keedle, Yu Gao, Jacqueline A. Boyle, Kingsley Agho, Hannah G. Dahlen

**Affiliations:** 1 Molly Wardaguga Research Centre, School of Nursing and Midwifery, Charles Darwin University, Level 11, 410 Ann Street, Brisbane, QLD 4000, Australia; sue.kildea@cdu.edu.au (S.K.); yu.gao@cdu.edu.au (Y.G.); 2 Department of Pediatrics, University of British Columbia, Vancouver, BC V6H 0B3, Canada; mia.mclean@bcchr.ca; 3 BC Children’s Hospital Research Institute, Vancouver, BC V5Z 4H4, Canada; 4 Douglas Institute Research Centre, 6875 Boulevard LaSalle, Verdun, Montreal, QC H4H 1R3, Canada; suzanne.king@mcgill.ca; 5 Department of Psychiatry, McGill University, 1033 Avenue des Pins, Montreal, QC H3A 1A1, Canada; 6 School of Nursing and Midwifery, Western Sydney University, Sydney, NSW 2751, Australia; h.keedle@westernsydney.edu.au (H.K.); h.dahlen@westernsydney.edu.au (H.G.D.); 7 Eastern Health Clinical School, Monash University, Melbourne, VIC 3128, Australia; jacqueline.boyle@monash.edu; 8 School of Health Sciences, Western Sydney University, Sydney, NSW 2751, Australia; k.agho@westernsydney.edu.au

**Keywords:** perinatal depression, social support, COVID-19 pandemic, prenatal stress

## Abstract

The COVID-19 pandemic has impacted perinatal mental health globally. We determined the maternal factors and pandemic-related experiences associated with clinically significant perinatal (pregnant and post-partum) depressive symptoms in Australian women. Participants (*n* = 2638; pregnant *n* = 1219, postnatal *n* = 1419) completed an online survey (August 2020 through February 2021) and self-reported on depression, social support, and COVID-19 related experiences. We found elevated depressive symptoms amongst 26.5% (pregnant) and 19% (postnatal) women. Multiple logistic regression analyses showed higher likelihood of elevated depression associated with residence in Victoria, lower education, past/current mental health problems, greater non-pandemic prenatal stress, age ≥ 35 years (pregnant women) and existing physical health issues or disability in self or others (postnatal women). Greater family stress/discord and lower social support (friends) was associated with higher odds of elevated perinatal depression, while lower social support (family) was significantly associated with elevated depressive symptoms in pregnant women. Greater depression was associated with social distancing, pandemic-related news exposure and changes to prenatal care (pregnant women). Single postnatal women showed lower odds of elevated depression than partnered women. Our findings underscore the importance of universal screening for depression and targeted support during a pandemic for perinatal women displaying vulnerability factors.

## 1. Introduction

Depression during pregnancy and in the first year post-birth (“perinatal depression”) is a public health concern due to its association with altered mother-infant interactions [1], more negative child developmental outcomes [2,3], and considerable personal and economic costs [4]. Stress during pregnancy is associated with increased risk of perinatal depression [5], hence, the increased stress and mental health impact of the COVID-19 pandemic globally [6] is of particular concern for perinatal mental health. Women may be more vulnerable to the mental health impacts of the pandemic during pregnancy [7], with elevated symptoms of depression reported in cross-sectional [8,9,10,11] and longitudinal research [12,13,14], together with studies comparing pre- and post-pandemic samples [15,16]. Rates of clinically significant self-reported depressive symptoms vary markedly across studies, from 15% in a multinational survey in June/July 2020 in the UK and Europe [17] to 37% in a study of Canadian pregnant women in April 2020 [11], likely due to the timing and local context of assessment. 

Previous studies have found that maternal sociodemographic factors associated with perinatal depressive symptoms during pre-pandemic times [18] may also increase vulnerability during the pandemic, including younger maternal age [8,17], lower education [16,19], and lower socio-economic status [8,20]. Consistent with pre-pandemic research, women with pre-existing physical or mental health concerns have demonstrated increased depressive symptoms during the pandemic [8,10,17,21], as have women with prenatal medical risk factors in many [8,16,20], but not all [21] studies. Conversely, greater social support has been found to be associated with lower depression, particularly for women who appraised the pandemic as more negative [19].

Women pregnant during the pandemic have been exposed to hardships that may increase vulnerability to mental health difficulties. Previous studies have reported higher depressive symptoms among pregnant women who report having a family member or friend infected with COVID-19 [21], are “in danger” of infection [19], or who live in geographical areas with a greater number of cases or deaths [16,22]. Even those living away from high exposure areas may experience perceived threat due to media exposure [23]. Loss of employment during the pandemic was associated with greater perinatal depression in some [8], but not all studies [11]. Women have experienced prenatal care changes such as fewer face-to-face appointments, and restrictions on partner and support people’s attendance at appointments, ultrasounds, or the birth in some cases [11,24]. Studies have found that changes to care during COVID-19 [25], and concerns about those changes [11], were predictors of depression in perinatal women. Lockdown and quarantine may impact on emotional wellbeing through a range of mechanisms, including increased relationship stress and reduced satisfaction in couple relationships [26,27] and increased social isolation and loneliness [11,14,19]. 

### 1.1. The Australian Context

After the World Health Organisation declared the novel coronavirus pandemic in March 2020 [28], Australia moved relatively quickly to adopt containment measures, including closing national borders and a national lockdown to combat the first wave of the COVID-19 outbreak from March to May 2020 [29]. A second wave in July 2020 saw the state of Victoria enter an additional 16 week lockdown [29]; among the longest continuous lockdowns in the world at the time [30]. Australia’s early response was considered to be among the most effective in the world for controlling transmission and minimising mortality due to COVID-19 [31]; however, the economic, social and personal impacts of these policies are considerable [32]. No published data currently exist on the risk factors for clinically significant depressive symptoms in perinatal women in Australia during COVID-19. To protect the wellbeing of perinatal women, it is essential to understand the size of the problem and factors that may identify who is at greatest need for support during COVID-19 and future pandemics in Australia. 

### 1.2. The Current Study

The Birth in the Time of COVID-19 Study (BITTOC) is a prospective longitudinal study of the impact of the COVID-19 pandemic on the mental health and wellbeing of Australian pregnant and birthing women and their infants. The current study aims to explore the factors (sociodemographic, psychosocial, prenatal, and pandemic-related factors) associated with greater perinatal depressive symptoms during the pandemic in Australian (1) pregnant and (2) postnatal women. 

## 2. Materials and Methods

### 2.1. Participants and Procedure

The current study included English-speaking Australian women over 18 years of age who were pregnant with singletons at any time during the first and second waves of the COVID-19 pandemic in Australia (i.e., from 20 March 2020, when Australian international borders closed). A convenience sample, recruited through advertisements on social media and Australian maternity and parenting websites, completed an online survey (August 2020 to February 2021). Informed consent was obtained from all participants online prior to commencement of the survey. Participants who completed the survey and provided a current email address entered a draw to win one of 100 gift vouchers worth $30. A total of 2638 (prenatal *n* = 1219, postnatal *n* = 1419) participants provided valid data for the current study. Ethical approval was received from Western Sydney University (#H13825) and Charles Darwin University (#H21052).

### 2.2. Measures

#### 2.2.1. Outcome: Maternal Depression

The Edinburgh Postnatal Depression Scale (EPDS) is a 10-item scale developed to assess symptoms of depression during the postnatal period [33], validated for use prenatally [34,35,36]. The EPDS has a maximum score of 30 and shows good psychometric properties [33], including with women from culturally and linguistically diverse backgrounds [37]. In the current study, scores ≥ 13 reflected clinically significant depressive symptoms [33,36].

#### 2.2.2. Sociodemographic Factors

Demographics gathered included: age, income, relationship status, state or territory of residence, country of birth and ethnic background, Aboriginal or Torres Strait Islander status and language spoken at home. 

#### 2.2.3. Psychosocial Factors

Participants reported on their current or previous mental health problems. Participants completed a modified version of the Negative Life Events Scale [38,39,40], inviting them to indicate whether any of 19 potentially stressful events (e.g., “divorce or separation”) and social health issues (e.g., “housing issues”) had been “a problem or worry for you or anyone close to you during this pregnancy”. Participants responded to a further item asking about their experience of three additional worries during their pregnancy (e.g., “worries there could be something wrong with baby”). Two scores were generated from these items: one score was allocated based on the number of potentially stressful events, social health issues, or pregnancy-related worries affecting the participant or her close contacts during pregnancy (“Non-Pandemic Stress”; maximum score = 19), and a separate score quantified the number of physical health concerns (“serious health issue”, “serious disability” or “other health worries”) affecting the participant or her close contacts during her pregnancy (“Pre-existing Health or Disability (self/close others)”; maximum score = 3). 

The Multidimensional Scale of Perceived Social Support (MSPSS; [41]) is a 12-item measure of perceived social support with three subscales: family, friends, and significant other. Items are answered using a 7-point Likert scale (1 = very strongly disagree, 7 = very strongly agree). Subscale scores were calculated using the mean of item responses, as recommended by the author [42]; higher scores represent greater social support. The MSPSS has demonstrated good reliability and validity [41], including in perinatal cohorts [43,44]. 

#### 2.2.4. Prenatal Factors

Participants provided information regarding parity, presence of prenatal medical risk factors, gestation at time of survey completion (pregnant participants) measured as first trimester (1–13 weeks), 2nd trimester (14–27 weeks) or 3rd trimester (28–41 weeks). Baby’s age at time of survey completion was reported by postnatal participants.

#### 2.2.5. COVID-19 Experiences

Participants completed an instrument developed to assess objective hardship due to COVID-19 (BITTOC Assessment of Stress Due to COVID; BASC150) during pregnancy. Based on scales used in previous research with pregnant women exposed to natural disasters [45,46], items assessed an individual’s experience of objective hardship with three main subscales: Threat of infection, Financial loss and Change to daily life (Non-pregnancy and Pregnancy-related), with each category worth a maximum of 50 points. Higher scores indicate greater objective hardship, with negative scores reflecting improvements due to the pandemic. Subscale scores may be summed to create a total objective hardship score (“BASC150”), with subscales examined separately in this study. A separate scale assessed degree of experience of social distancing (e.g., reduced in-person contact, activities cancelled or avoided), giving a total score of 40 (“Social distancing”). The “Scope” of the pandemic was calculated for each participant according to the date they completed the survey, including the duration and intensity of the COVID-19 crisis at the time, based on the participant’s state of residence. Refer to Supplementary Table S1 for further details on these measures. Participants also completed items assessing the number of hours spent each day consuming news about COVID-19 (TV reports, newspaper articles, podcasts, radio, or online news) and the degree of stress and discord experienced within the family attributed to the pandemic. As participants reported on their experiences of the pandemic during pregnancy, those who were recruited postnatally provided retrospective reports. 

### 2.3. Statistical Analysis

Data were analysed using SPSS v26.0 [47]. Data were screened for potential bots and fraudulent responses based on several criteria [48,49] and cases meeting multiple criteria and records with <50% of the survey completed were excluded (further details may be obtained from study authors). The dichotomised depression variable (1 = elevated depressive symptoms, 0 = non-elevated depressive symptoms) was examined against a set of potential risk factors (sociodemographic, psychosocial, prenatal and COVID-19 related). Univariate logistic regression analyses were conducted to determine the relationship between the outcome and each independent variable. Based on the results of the univariate logistic regression analyses, all factors with *p* < 0.1 were retained and were used to build a multiple binomial logistic regression model to determine the unique association of each variable with the outcome whilst controlling for other factors. “Crude” and “adjusted” odds ratios, with their corresponding 95% CIs, were calculated to assess the odds in the univariate logistic regression and multiple logistic regression models, respectively. Analyses included cases with complete data.

#### Missing Data

Comparison of valid cases who completed the EPDS (*n* = 2638) with valid cases missing EPDS data (*n* = 330) indicated that the latter were more likely to report a family income < $100,000 (χ^2^(1) = 11.93, *p* = 0.001), less likely to be university educated (χ^2^(1) = 28.45, *p* < 0.001), and less likely to report being affected by a physical health problem or disability concerning themselves or a close other χ^2^(1) = 6.47, *p* = 0.011). Of those participants who completed the full survey, including the EPDS, analysis of missing items indicated that data were missing completely at random (MCAR) for both pregnant (Little’s Missing Completely at Random (MCAR test), χ^2^ (28) = 27.45, *p* = 0.494) and postnatal participants (Little’s MCAR test, χ^2^ (28) = 30.91, *p* = 0.321). Missing data were handled with listwise deletion.

## 3. Results

### 3.1. Participant Characteristics

Participant characteristics (Table 1) and descriptive statistics for COVID-19 objective hardship variables are presented (Table 2). Most (85%) study participants were born in Australia, and a majority resided in New South Wales and Victoria. Compared to the distribution of births in Australia [50], our cohort showed some overrepresentation in New South Wales and Victoria, and some underrepresentation in Queensland and Western Australia. All analyses examined NSW and Victoria separately, combining the remaining states and territories (*n* = 808). The most frequent maternity care providers were private obstetrician (25.8%; *n* = 680), public hospital maternity care (25.5%, *n* = 673), and midwifery group practice (18.4%; *n* = 486). Compared with the Australian population [51], our sample included fewer consumers of public hospital maternity care (40.8%), and greater use of private obstetric care (11%). Five participants reported that they tested positive for COVID-19 during their pregnancy, and 162 pregnant (13.2%) and 125 postnatal women (8.8%) reported having a friend or family member who had returned a positive test result. Current mental health treatment was reported by 18.1% (*n* = 220) of pregnant women and 15.8% (*n* = 224) of postnatal women, with past mental health treatment reported by 27.1% (*n* = 329) of pregnant women and 22.7% (*n* = 322) of postnatal women. Postnatal women reported that their baby was aged between 0 and 32 weeks (M = 10.92, SD = 5.82) at the time of survey completion.

Mean (SD) scores for depressive symptoms in the pregnant and postnatal cohorts were 8.73 (5.51) and 7.68 (5.53), respectively. Overall, 22% (*n* = 581) of study participants scored above the clinical cut-off on the EPDS signalling clinically significant depressive symptoms. Of those scoring in the elevated range, 30.9% (*n* = 96) of pregnant women and 39% (*n* = 105) of postnatal women reported current mental health treatment. Of those in the normal range on the EPDS, 13.7% (*n* = 124) of pregnant women and 10.4% (*n* = 119) of postnatal women were receiving mental health treatment.

### 3.2. Aim 1: Factors Associated with Elevated Depression in Pregnant Women

After conducting univariate logistic regressions to examine potential risk factors for depression in the pregnant cohort, associated factors (*p* > 0.1) were entered into the final, multivariable model to determine unique associations of each factor with the outcome when other factors were accounted for (Table 3). In this model, elevated depressive symptoms were significantly associated with residence in Victoria, older maternal age (≥35 years), education below university level, past/current mental health problems, and reporting more sources of non-pandemic stress during pregnancy. Pregnant women who perceived greater social support from family and friends were less likely to report depressive symptoms in the elevated range. The COVID-19 objective hardships significantly associated with elevated depressive symptoms were changes to prenatal care, engaging in more social distancing, consuming more pandemic-related news, and reporting mild to severe family stress/discord. 

### 3.3. Aim 2: Factors Associated with Elevated Depression in Postnatal Women 

Factors with associations (*p* > 0.1) with elevated depression in the postnatal cohort in univariate logistic regressions (Table 4) were entered into the final, multiple logistic regression model. Elevated depression amongst postnatal women was associated with residing in Victoria, education below university level, past/current mental health problems, being affected by a physical health problem or disability in self or close others, reporting more sources of non-pandemic stress during pregnancy. Women reporting no current partner had lower odds of depression, as did those reporting greater perceived social support from friends, while mild to severe family stress/discord due to the pandemic was associated with elevated depressive symptoms. 

## 4. Discussion

The current study is the first to our knowledge to report on the correlates of depressive symptoms in Australian perinatal women during the COVID-19 pandemic. Over 25% of pregnant women and 19% of postnatal women reported clinically significant symptoms of depression. Risk and protective factors were identified that may influence the vulnerability of perinatal women to depression during the pandemic. Several types of COVID-19 experiences were significantly associated with elevated depressive symptoms, particularly increased family stress/discord in both pregnant and postnatal women, but also changes to prenatal care, social distancing, and pandemic-related news consumption in pregnant women. 

The proportion of elevated depressive symptoms in the perinatal women of the current study was approximately two to three times greater than point-prevalence estimates using the same measure in Australian community samples pre-COVID-19, that is, between 5.9% and 6.2% (prenatal), and 3.3% and 8.8% (postnatal) [18,52,53,54]. Despite experiencing relatively low rates of COVID-19 cases and deaths at the time of assessment, the proportions we report here exceed the 15% (pregnant) and 13% (up to 3 months postnatal, breast-feeding mothers only) reported in a multinational study in Europe and the UK [17], but are lower than the 33.2% in postnatal (0 to 18 months) women in Canada [55] and 34.2% (pregnant) and 26.3% (postnatal) women in Italy [25]. Variability in rates of elevated depression during the pandemic may reflect the influence of location (proximity to major outbreaks) and timing of assessment, with studies conducted earlier in the pandemic reporting greater rates of depression compared with those found here [11,56]. Those surveyed when restrictions were being eased showed lower proportions than ours [17]. While not the aim of the current study, it is interesting to note that a significantly greater proportion of pregnant women reported elevated depressive symptoms compared with postnatal women, however, they were also more likely to report current or previous mental health problems, hence may have had greater vulnerability to pandemic-related distress or were already seeking support for mental health problems arising from the pandemic. 

It is noteworthy that 60 to 70% of women reporting clinically significant depressive symptoms were not receiving any form of mental health treatment at the time of the survey, suggesting that alarming numbers of women are experiencing significant distress that has gone undetected and unsupported. While considerable progress has been made in the detection of perinatal depression in Australia through the implementation of universal screening during pregnancy [36], 16.9% of Australian women reported not being asked about their mental health at all during the perinatal period [57]. Screening rates continue to lag in the private sector [58], with the EPDS completed by only 28.8% of women receiving private maternity care in 2015 in Queensland, compared with 91.1% of public patients [59]. Considering the impact of untreated mental health problems for women and their infants [2,60], improving the support for perinatal women in distress during a pandemic is of critical public health importance.

### 4.1. Sociodemographic Factors

Certain risk factors for elevated depression were common across both cohorts. Living in the state of Victoria was associated with three to four times the odds of elevated depression. Although models controlled for scope of pandemic exposure (local case/death numbers, and duration) and numerous other COVID-19 related objective hardships, Victoria experienced a rapid increase in cases and additional lockdowns and maternity care restrictions due to a second wave outbreak just prior to survey administration. The additional distress experienced by Victorian women, seen here, may reflect this experience. 

Greater maternal age (≥35 years) was independently associated with elevated prenatal depressive symptoms during the pandemic. This conflicts with some pandemic studies finding greater risk of depression amongst younger women [17], but is consistent with pandemic [8,19] and pre-pandemic studies showing greater risk for perinatal depression with increased maternal age [18]. Lower income was, in univariate analyses, associated with elevated depression. However, income was no longer associated with depression when considering it in the multivariable model, in conjunction with other risk factors. Instead, pregnant women were more likely to report elevated depression if they had lower education, consistent with previous studies [16,19], with a similar effect for postnatal women that fell short of statistical significance in the multivariable model. Given that both income and education were entered into the multivariable model, it is possible that education confounded the significant association detected between income and elevated depression in the univariate analyses. Furthermore, with the influence of income adjusted for in the multivariable models, it is possible that the role of education may reflect patterns in occupation, with women with lower education possibly employed in industries more impacted by public health directives, such as the service sector where women were either in greater contact with people or were more likely to be laid off [61]. 

A surprising finding concerned relationship status; while women reporting single status showed increased odds of depression in unadjusted models, single postnatal women showed reduced odds of reporting elevated depression once sociodemographic, psychosocial, prenatal, and pandemic-related factors were covaried. While pre-pandemic studies show increased depression symptoms amongst single mothers, this has been shown to be accounted for by socioeconomic and psychosocial correlates, such as financial hardship and low social support [62,63]. It is possible that, when well-resourced and receiving adequate social support in other ways, single mothers in our study may have possessed other psychological or personality characteristics (e.g., resilience; [64]), reducing their risk of poor postnatal wellbeing during the pandemic.

### 4.2. Psychosocial Factors

Both pregnant and postnatal women reporting current or past mental health problems were more likely to score in the elevated range on the EPDS, consistent with previous studies of perinatal women during the COVID-19 pandemic [8,10,17,21], and pre-pandemic studies of the aetiology of perinatal depression [5]. Similarly, women reporting greater non-pandemic stressful life events and social health issues during pregnancy were more likely to report increased depression, which aligns with findings outside of the pandemic context [5,65]. 

Perinatal women may live with additional challenges that were exacerbated by the pandemic. Postnatal women managing (or supporting a loved one with) a pre-existing health issue or disability showed higher odds of elevated depression, consistent with other studies [21]. This may reflect increased concern about threat of infection for those at risk of severe illness from COVID-19, the disruption to routine medical or psychosocial care, or the additional burden of managing physical health or disability issues in the family when also caring for a newborn, with reduced access to external support. Conversely, perinatal women showed reduced risk of depression if they reported greater perceived social support, consistent with Lebel, MacKinnon, Bagshawe, Tomfohr-Madsen and Giesbrecht [11] and pre-pandemic research [5]. We extended previous studies to demonstrate that support specifically from friends was associated with reduced likelihood of elevated depression during the perinatal period [66]. In pregnant women, greater perceived social support from family was also associated with lower depression risk, which may reflect the importance of perceived availability of practical support when pregnant during the pandemic [65]. 

### 4.3. Prenatal Factors

Previous studies have found an association between prenatal medical risk factors and perinatal depression risk during COVID-19 [8,16,20]. In the current cohort, perinatal women who self-reported increased prenatal medical risk were more likely to endorse elevated depression in unadjusted analyses. However, these associations were no longer evident in the multivariable model, consistent with the findings reported by Zeng, Li, Sun, Luo, Garg, Liu, Zhang and Zhang [21]. Similarly, postnatal women with prenatal medical factors who had older babies at the time of survey completion showed higher rates of depression in the unadjusted, but not multivariable model. It is possible that these prenatal risk factors may have been moderated by other pandemic hardships, including changes to prenatal care.

### 4.4. COVID-19 Experiences

There are multiple mechanisms by which the COVID-19 pandemic may influence perinatal mental health [7], and we sought to understand the objective hardships with most impact for the development of depression. The most influential pandemic-related factor predicting outcomes in this study was family stress/discord. Pregnant women reporting moderate to severe household discord (e.g., people frequently short-tempered with one another, physical fights amongst children or adults, throwing or knocking over property) demonstrated five times the odds of elevated depression as those reporting no stress/discord. However, even mild increases in family stress/discord were associated with greater depression risk in both cohorts, corresponding with studies of perinatal women identifying a role of relationship strain for predicting depression risk during COVID-19 [11], and pre-pandemic associations between depressive symptoms and both intimate partner violence [18,67] and poor relationship quality [68,69]. COVID-19 has been associated with worse mental health in the adult population generally [70], and partners of women experiencing perinatal depression are more likely to develop their own mood disturbance [71]. Together, these findings highlight the importance of considering pandemic stress in the family, including the potential role of couple interventions to reduce the risk of perinatal depression in vulnerable families [72]. 

Direct exposure to COVID-19, through greater scope (intensity and duration) of exposure in the geographical area at the time of survey completion was not associated with greater depressive symptoms. Similarly, the association between depression and threat of COVID-19 infection (i.e., symptoms, testing and diagnosis) in self or close others fell short of statistical significance, contrasting with previous studies [16,21], which may be due to the comparatively low COVID-19 incidence in Australia at the time. However, indirect exposure to COVID-19 through pandemic-related news consumption was associated with greater risk of depression in pregnant women, even after adjusting for actual exposure to cases, consistent with Basu, Kim, Basaldua, Choi, Charron, Kelsall, Hernandez-Diaz, Wyszynski and Koenen [9]. Olagoke, Olagoke and Hughes [23] also found that greater engagement with pandemic-related news was associated with increased depression in adults, with this association mediated by increased perceived vulnerability to COVID-19. Accordingly, women for whom the actual threat of COVID-19 infection is low may have experienced heightened perceived threat through news exposure, resulting in worse mental health outcomes. Together, these findings suggest that reducing uptake of pandemic-related news may be a promising behavioural intervention for reducing risk of depression in vulnerable perinatal women. 

The potential adverse mental health impacts of social distancing during the pandemic [70] was found again here. Social distancing may impact on the ability of families to engage in rituals around the transition to motherhood, such as baby showers [73,74], and changed routines may have impacted on women’s engagement with positive health behaviours, such as exercise and accessing social support [75,76,77]. Furthermore, pregnant women reporting more changes to their prenatal care (e.g., reduced or cancelled appointments) had a higher likelihood of elevated depression, which is consistent with other studies [11]. Wilson*,* et al. [78] found that Australian pregnant women reported increased isolation and reduced autonomy around their maternity care experience due to health service restrictions, which may exacerbate the distress experienced by women during a particularly vulnerable time. 

While these COVID-19 experiences were found to be statistically significant for predicting depression status, particularly in prenatal women, it is important to note that some effect sizes were small. While this may be due to Australia being relatively less affected in terms of COVID-19 incidence compared with other countries, it is also likely that the association between pandemic-related objective hardship and mental health outcomes is mediated or moderated by other maternal psychological characteristics, such as resilience or cognitive appraisal [19], or maternity care [79]. Further research will assist to understand the complex pathways predicting maternal outcomes during the pandemic, hence opportunities for prevention and intervention.

### 4.5. Strengths and Limitations

Our study benefited from a large sample, with participants from all states and territories of Australia, providing the opportunity for a comprehensive model of factors to be tested, including both pandemic-related experiences and established vulnerability factors for perinatal depression during pre-pandemic times. Our COVID-19 objective hardship variables were developed for the Australian context based on previous disaster research with perinatal women [45], and the scope variable enabled the COVID-19 exposure of a geographical area to be quantified, with the pandemic experience likely to be influenced by each unique local context. Despite these strengths, the present findings should be interpreted with caution. Firstly, as a cross-sectional survey, it is not possible to infer causality based on the associations found. Being an online survey, women self-selected into the study, and were predominantly White, spoke English and had higher income, limiting the generalisability of results to more diverse populations who may have less access to, or comfort with, the use of technology. Conversely, our cohort had a relatively high rate of current and previous mental health problems, which may suggest that more vulnerable women have self-selected into the study. Future research with random samples, including individuals from diverse cultural and linguistic backgrounds, will be important to determine the generalizability of the current findings. The postnatally recruited women completed the survey concerning prenatal COVID-19 experiences up to eight months post-birth, hence recall bias cannot be ruled out. We also cannot exclude the influence of residual confounding on the current results.

## 5. Conclusions

Our findings provide insight into the factors associated with elevated depression during the perinatal period in Australian women. Results underscore the importance of screening and monitoring of depressive symptoms in perinatal women, including assessment for associated sociodemographic and psychosocial factors to facilitate reliable detection of vulnerable women. Particular attention should be given to women residing in Victoria, those with lower education levels, greater non-pandemic stress during pregnancy, previous mental health issues, and women managing physical health or disability issues in themselves or family members. Further research is needed to explore the moderators of maternal wellbeing during COVID-19, including the psychological characteristics and maternity care practices that may buffer the adverse effects of pandemic-related impacts on women’s mental health and wellbeing.

## Figures and Tables

**Table 1:** Characteristics of study participants.

	Pregnant (*n* = 1219)	Postnatal (*n* = 1419)
	*N* (Valid %) or M (SD)	*N* (Valid %) or M (SD)
**Sociodemographic factors**		
State/Territory in Australia		
New South Wales	447 (36.7)	560 (39.9)
Victoria	438 (36.0)	369 (26.3)
Queensland	169 (13.9)	230 (16.4)
Western Australia	55 (4.5)	94 (6.7)
South Australia	53 (4.4)	78 (5.6)
Australian Capital Territory	30 (2.5)	27 (1.9)
Tasmania	21 (1.7)	30 (2.1)
Northern Territory	5 (0.4)	16 (1.1)
Maternal age		
<35 years old	917 (75.2)	1033 (72.8)
≥35 years old	302 (24.8)	386 (27.2)
Relationship status		
Current partner	1193 (97.9)	1361 (96.8)
No current partner	26 (2.1)	45 (3.2)
Income		
<$100,000	349 (30.3)	399 (30.2)
≥$100,000	803 (69.7)	923 (69.8)
University education		
No	386 (31.7)	457 (32.2)
Yes	833 (68.3)	962 (67.8)
Aboriginal or Torres Strait Islander		
No	1192 (98.5)	1382 (98.6)
Yes	18 (1.5)	19 (1.4)
Language spoken at home		
English	1085 (89.0)	1297 (92.2)
Non-English	134 (11.0)	109 (7.08)
**Psychosocial factors**		
Past/Current mental health problems		
No past/current MH problems	602 (49.4)	807 (56.9)
Past/current MH problems	617 (50.6)	612 (43.1)
Pre-existing health or disability (self/close others)		
No pre-existing health or disability issues	978 (80.2)	1202 (84.7)
Pre-existing health or disability issues	241 (19.8)	217 (15.3)
Non-pandemic stress ^a^	3.15 (2.31)	2.50 (2.06)
Perceived social support ^b^		
Significant other	6.25 (1.16)	6.22 (1.21)
Family	5.72 (1.32)	5.78 (1.32)
Friends	5.50 (1.34)	5.62 (1.34)
**Prenatal and infant factors**		
Parity		
Primiparous	443 (36.3)	660 (46.9)
Multiparous	776 (63.7)	746 (53.1)
Prenatal medical risk factors		
No risk factors	730 (59.9)	714 (50.3)
Mild/serious RFs	489 (40.1)	705 (49.7)
Gestation at time of survey		
First trimester (1–13 weeks)	104 (8.5)	
Second trimester (14–27 weeks)	476 (39)	
Third trimester (28–41 weeks)	639 (52.4)	
Baby’s age at time of survey (weeks)		10.92 (5.82)

Note: M = Mean; SD = Standard deviation. ^a^ Number of non-pandemic prenatal stressful life events, social health issues or pregnancy-related worries; ^b^ Multidimensional Scale of Perceived Social Support.

**Table 2:** Descriptive statistics for depression and COVID Experiences.

	Pregnant				Postnatal			
	*N*	M	SD	Range	*N*	M	SD	Range
Scope	1204	30.25	2.95	16–43	1394	29.94	2.83	16–46
Threat of infection	1219	2.73	4.32	0–33	1419	1.32	3.27	0–23
Financial loss	1219	10.95	11.23	−4–50	1419	9.66	11.13	−4–50
Change (Non-pregnancy)	1219	6.97	3.40	0–15	1419	5.92	3.38	0–15
Change (Pregnancy-related)	1219	7.01	4.82	−1–26	1419	6.38	4.57	−2–32
Social distancing	1219	29.67	6.17	0−40	1419	29.35	6.02	0–40
COVID-19 news exposure	1219	2.01	1.63	0−16	1419	2.49	1.95	0–20

Note: M = Mean; SD = Standard deviation.

**Table 3:** Pregnant women: factors associated with elevated depressive symptoms (EPDS ≥ 13).

			Univariate Logistic Regression		Multiple Logistic Regression (*n* = 1116)	
	EPDS < 13 N (%)/ M (SD)	EPDS ≥ 13 N (%)/ M (SD)	cOR (95% CI)	*p* Value	aOR (95% CI)	*p* Value
**Sociodemographic factors**						
State/Territory in Australia						
All other states	272 (81.7)	61 (18.3)	Ref.		Ref.	
NSW	364 (81.4)	83 (18.6)	1.02 (0.71, 1.47)	0.929	1.04 (0.64, 1.69)	0.882
VIC	271 (61.9)	167 (38.1)	**2.75 (1.96, 3.85)**	**<0.001**	**2.97 (1.75, 5.05)**	**<0.001**
Maternal age						
<35 years old	695 (75.8)	222 (24.2)	Ref.		Ref.	
≥35 years old	212 (70.2)	90 (29.8)	1.33 (1.00, 1.78)	0.054	**1.65 (1.11, 2.46)**	**0.014**
Relationship status						
Current partner	893 (74.9)	300 (25.1)	Ref.			
No current partner	14 (53.8)	12 (46.2)	**2.55 (1.17, 5.58)**	**0.019**	0.98 (0.32, 2.97)	0.964
Income						
≥$100,000	626 (78.0)	177 (22.0)	Ref.			
<$100,000	238 (68.2)	111 (31.8)	**1.65 (1.25, 2.18)**	**<0.001**	0.79 (0.53, 1.17)	0.242
University education						
No	256 (66.3)	130 (33.7)	Ref.		Ref.	
Yes	651 (78.2)	182 (21.8)	**0.55 (0.42, 0.72)**	**<0.001**	**0.56 (0.38, 0.82)**	**0.003**
Aboriginal or Torres Strait Islander						
No	888 (74.5)	304 (25.5)	Ref.			
Yes	14 (77.8)	4 (22.2)	0.84 (0.27, 2.56)	0.751		
Language spoken at home						
English	805 (74.2)	280 (25.8)	Ref.			
Non-English	102 (76.1)	32 (23.9)	0.90 (0.59, 1.37)	0.902		
**Psychosocial factors**						
Past/current mental health problems						
No	501 (83.2)	101 (16.8)	Ref.		Ref.	
Yes	406 (65.8)	211 (34.2)	**2.58 (1.97, 3.38)**	**<0.001**	**1.93 (1.35, 2.78)**	**<0.001**
Pre-existing health or disability (self/others)						
No	768 (78.5)	210 (21.5)	Ref.			
Yes	139 (57.7)	102 (42.3)	**2.68 (1.99, 3.61)**	**<0.001**	1.41 (0.93, 2.13)	0.108
Non-pandemic stress ^a^	2.67 (2.05)	4.54 (2.43)	**1.43 (1.34, 1.52)**	**<0.001**	**1.24 (1.14, 1.35)**	**<0.001**
Perceived social support ^b^						
Significant other	6.39 (1.08)	5.84 (1.28)	**0.69 (0.62, 0.77)**	**<0.001**	0.98 (0.81, 1.19)	0.845
Family	5.91 (1.22)	5.15 (1.41)	**0.67 (0.61, 0.74)**	**<0.001**	**0.79 (0.66, 0.94)**	**0.009**
Friends	5.70 (1.26)	4.93 (1.41)	**0.67 (0.61, 0.74)**	**<0.001**	**0.78 (0.67, 0.91)**	**0.002**
Prenatal factors						
Parity						
Primiparous	330 (74.5)	113 (25.5)	Ref.			
Multiparous	577 (74.4)	199 (25.6)	1.01 (0.77, 1.32)	0.958		
Prenatal medical risk factors						
No risk factors	573 (78.5)	157 (21.5)	Ref.			
Mild/Serious RFs	334 (68.3)	155 (31.7)	**1.69 (1.31, 2.20)**	**<0.001**	1.37 (0.97, 1.94)	0.074
Gestation						
1st trimester	78 (75.0)	26 (25.0)	0.91 (0.57, 1.47)	0.706		
2nd trimester	361 (75.8)	115 (24.2)	0.87 (0.66, 1.15)	0.325		
3rd trimester	468 (73.2)	171 (26.8)	Ref.			
**COVID-19 experiences**						
Scope	30.89 (2.89)	30.73 (3.07)	**1.07 (1.03, 1.12)**	**<0.001**	0.98 (0.91, 1.04)	0.480
Threat of infection	2.45 (4.12)	3.54 (4.79)	**1.06 (1.03, 1.09)**	**<0.001**	1.04 (1.00, 1.07)	0.063
Financial loss	9.85 (10.65)	14.15 (12.25)	**1.03 (1.02, 1.05)**	**<0.001**	1.00 (0.99, 1.02)	0.660
Change (Non-pregnancy)	6.75 (3.40)	7.63 (3.34)	**1.08 (1.04, 1.12)**	**<0.001**	1.02 (0.97, 1.08)	0.458
Change (Pregnancy-related)	6.34 (4.42)	8.97 (5.37)	**1.12 (1.09, 1.15)**	**<0.001**	**1.05 (1.01, 1.09)**	**0.007**
Social distancing	29.26 (6.17)	30.86 (6.05)	**1.05 (1.02, 1.07)**	**<0.001**	**1.04 (1.01, 1.08)**	**0.014**
COVID-19 news exposure	1.84 (1.41)	2.49 (2.09)	**1.25 (1.16, 1.35)**	**<0.001**	**1.15 (1.04, 1.27)**	**0.009**
Family stress/discord						
None	470 (88.3)	62 (11.7)	Ref.		Ref.	
Mild	397 (68.1)	186 (31.9)	**3.55 (2.59, 4.88)**	**<0.001**	**2.40 (1.61, 3.57)**	**<0.001**
Moderate—Severe	31 (35.6)	56 (64.4)	**13.69 (8.2, 22.86)**	**<0.001**	**5.32 (2.81, 10.08)**	**<0.001**

Note: M = Mean; SD = Standard deviation; cOR = crude odds ratios for univariate logistic regressions; aOR = adjusted odds ratio for independent variable when all other variables *p* < 0.1 are retained in the multivariable model, i.e., state/territory in Australia, maternal age, relationship status, income, university education, past/current mental health problems, pre-existing health or disability (self/others), non-pandemic prenatal stressful life events, social health issues or pregnancy-related worries, perceived social support, prenatal medical risk factors, COVID-19 Scope, Threat of infection, Financial loss, Change (Non-pregnancy), Change (Pregnancy-related), Social distancing, COVID-19 news exposure, Family stress/discord; CI = confidence interval. Numbers in bold represent significant associations (*p* < 0.05). Depressive symptoms assessed by the Edinburgh Postnatal Depression Scale (EPDS) were dichotomised with category 1 for elevated depressive symptoms and category 0 for non-elevated depressive symptoms. ^a^ Number of non-pandemic prenatal stressful life events, social health issues or pregnancy-related worries; ^b^ Multidimensional Scale of Perceived Social Support.

**Table 4:** Postnatal women: factors associated with elevated depressive symptoms (EPDS ≥ 13).

			Univariate Logistic Regression		Multiple Logistic Regression (*n* = 1273)	
	EPDS < 13 N (%)/M (SD)	EPDS ≥ 13 N (%)/M (SD)	cOR (95% CI)	*p* Value	aOR (95% CI)	*p* Value
**Sociodemographic factors**						
State/Territory in Australia						
All other states	412 (86.7)	63 (13.3)	Ref.		Ref.	
NSW	473 (84.5)	87 (15.5)	1.20 (0.85, 1.71)	0.395	1.37 (0.90, 2.10)	0.146
VIC	254 (68.8)	115 (31.2)	**2.96 (2.10, 4.18)**	**<0.001**	**4.25 (2.58, 6.99)**	**<0.001**
Maternal age						
<35 years old	826 (80.0)	207 (20.0)	Ref.			
≥35 years old	324 (83.9)	62 (16.1)	0.76 (0.56, 1.04)	0.09	0.86 (0.70, 1.05)	0.133
Relationship status						
Current partner	1100 (81.6)	251 (18.4)	Ref.			
No current partner	31 (68.9)	14 (31.1)	**2.00 (1.05, 3.81)**	**0.036**	**0.23 (0.07, 0.76)**	**0.016**
Income						
≥$100,000	776 (84.1)	147 (15.9)	Ref.			
<$100,000	300 (75.2)	99 (24.8)	**1.74 (1.31, 2.32)**	**<0.001**	1.04 (0.71, 1.54)	0.831
University education						
No	344 (75.3)	113 (24.7)	Ref.		Ref.	
Yes	806 (83.8)	156 (16.2)	**0.59 (0.45, 0.77)**	**<0.001**	0.70 (0.48, 1.02)	0.061
Aboriginal or Torres Strait Islander						
No	1122 (81.2)	230 (18.8)	Ref.			
Yes	15 (78.9)	4 (21.1)	1.15 (0.38, 3.50)	0.804		
Language spoken at home						
English	1056 (81.4)	241 (18.6)	Ref.			
Non-English	85 (78.0)	24 (22.0)	1.24 (0.77, 1.99)	0.379		
**Psychosocial factors**						
Past/current mental health problems						
No	720 (89.2)	87 (10.8)	Ref.		Ref.	
Yes	460 (70.3)	182 (29.7)	**3.50 (2.64, 4.64)**	**<0.001**	**2.76 (1.96, 3.88)**	**<0.001**
Pre-existing health or disability (self/others)						
No	1005 (83.6)	197 (16.4)	Ref.		Ref.	
Yes	145 (66.8)	72 (33.2)	**2.53 (1.84, 3.49)**	**<0.001**	**1.60 (1.07, 2.41)**	**0.023**
Non-pandemic stress ^a^	2.21 (1.89)	3.74 (2.31)	**1.39 (1.31, 1.49)**	**<0.001**	**1.17 (1.07, 1.27)**	**<0.001**
Perceived social support ^b^						
Significant other	6.32 (1.16)	5.81 (1.32)	**0.75 (0.68, 0.82)**	**<0.001**	0.94 (0.79, 1.13)	0.534
Family	5.88 (1.31)	5.36 (1.26)	**0.77 (0.70, 0.84)**	**<0.001**	1.02 (0.86, 1.22)	0.796
Friends	5.76 (1.29)	5.04 (1.41)	**0.71 (0.65, 0.77)**	**<0.001**	**0.79 (0.68, 0.92)**	**0.003**
**Prenatal factors**						
Parity						
Primiparous	535 (81.1)	125 (18.9)	Ref.			
Multiparous	606 (81.2)	140 (18.8)	0.99 (0.76, 1.29)	0.934		
Prenatal medical risk factors						
No risk factors	599 (83.9)	115 (16.1)	Ref.			
Mild/serious RFs	551 (78.2)	154 (21.8)	**1.46 (1.11, 1.90)**	**0.006**	1.00 (0.72, 1.40)	0.987
Baby’s age (weeks)	10.76 (5.82)	11.59 (5.82)	**1.03 (1.00, 1.05)**	**0.036**	1.02 (0.98, 1.05)	0.347
COVID-19 experiences						
Scope	29.82 (2.83)	30.43 (2.74)	**1.08 (1.03, 1.13)**	**0.002**	0.98 (0.91, 1.05)	0.505
Threat of infection	1.21 (3.08)	1.79 (3.99)	**1.05 (1.01, 1.09)**	**0.01**	1.02 (0.98, 1.07)	0.322
Financial loss	8.98 (10.68)	12.57 (12.49)	**1.03 (1.02, 1.04)**	**<0.001**	1.01 (1.00, 1.03)	0.161
Change (Non-pregnancy)	5.84 (3.38)	6.26 (3.37)	1.04 (1.00, 1.08)	**0.063**	0.98 (0.93, 1.04)	0.477
Change (Pregnancy-related)	6.00 (4.26)	7.97 (5.44)	**1.09 (1.06, 1.12)**	**<0.001**	1.03 (0.99, 1.07)	0.107
Social distancing	29.23 (6.03)	29.84 (5.96)	1.02 (1.00, 1.04)	0.135		
COVID-19 news exposure	2.40 (1.86)	2.86 (2.25)	**1.11 (1.05, 1.18)**	**0.001**	1.05 (0.98, 1.14)	0.190
Family stress/discord						
None	587 (89.5)	69 (10.5)	Ref.		Ref.	
Mild	499 (76.5)	153 (23.5)	**2.61 (1.92, 3.55)**	**<0.001**	**1.71 (1.18, 2.47)**	**0.005**
Moderate to severe	53 (56.4)	41 (43.6)	**6.58 (4.08, 10.61)**	**<0.001**	**2.64 (1.41, 4.93)**	**0.002**

Note: M = Mean; SD = Standard deviation; cOR = crude odds ratios for univariate logistic regressions; aOR = adjusted odds ratio for independent variable when all other variables *p* < 0.1 are retained in the multivariable model, i.e., state/territory in Australia, maternal age, relationship status, income, university education, past/current mental health problems, pre-existing health or disability (self/others), non-pandemic prenatal stressful life events, perceived social support, prenatal medical risk factors, COVID-19 Scope, Threat of infection, Financial loss, Change (Non-pregnancy), Change (Pregnancy-related), COVID-19 news exposure, Family stress/discord; CI = confidence interval. Numbers in bold represent significant associations (*p* < 0.05). Depressive symptoms assessed by the Edinburgh Postnatal Depression Scale (EPDS) were dichotomised with category 1 for elevated depressive symptoms and category 0 for non-elevated depressive symptoms. ^a^ Number of non-pandemic prenatal stressful life events, social health issues or pregnancy-related worries; ^b^ Multidimensional Scale of Perceived Social Support.

## Data Availability

The data presented in this study are available on request from the corresponding author.

## Supplementary Materials

The following supporting information can be downloaded at: https://www.mdpi.com/article/10.3390/ijerph19095062/s1, Table S1: Description of scales assessing COVID-19 related experiences.
