# Supplementary material for: Perinatal Depression in Australian Women during the COVID-19 Pandemic: The Birth in the Time of COVID-19 (BITTOC) Study
Source: Int J Environ Res Public Health. 2022 Apr 21;19(9):5062. doi: 10.3390/ijerph19095062 (PMC9103175; doi:10.3390/ijerph19095062)
Supplement: Supplementary file 1 [file ijerph-19-05062-s001.zip › ijerph-1652806-supplementary.pdf]

Table S1. Description of scales assessing COVID-19 related experiences.

Scale	Description
Threat50	Assesses the level of threat of infection experienced due to COVID-19 reported by the respondent, and includes items indicating the degree to which the health of the respondent, or their family and friends, was threatened due to COVID-19 (e.g., COVID-19 symptoms and their severity). Comprised of 6 items with yes/no (e.g., *Have you received a positive result from a COVID-19 test?*) or multiple response items, weighted for severity of impact to obtain a maximum score of 50. For example, "If you believe that you have had COVID-19, how severe was it?", with response options scored from 0 (No symptoms) to 17 (*Severe. Symptoms severe and required ventilation (you would have been admitted in the intensive care unit (the ICU) with intubation*).
Loss50	Assesses the level of financial loss experienced by the respondent due to COVID-19, and includes items assessing any loss of income or savings, job security, or investments (3 items), and the degree to which this loss of income impacted their life, from "*Better than before*" to "*Severe. Unable to meet basic needs and/or pay bills*". Scoring of items is weighted according to degree of severity/disruption with a total maximum score of 50.
Change50	Assesses the amount of change in the daily life and pregnancy plans that the respondent experienced due to the COVID-19 crisis, including changes in daily and work routine, social distancing, pregnancy care support, pregnancy class and care practice, and birth plans, due to COVID-19. Incorporates two subscales:<br>- **Change (Non-pregnancy)** assesses which of five possible changes was experienced by the participant (e.g., "moved to remote work or working from home") along with the degree to which COVID-19 had changed their life in terms of daily routine, from 0 (*No change*) to 3 (*Severe. Change in three or more areas (e.g., work, education, social life, hobbies, religious activities).* Scoring is weighted to obtain a maximum total score of 15.<br>- **Change (Pregnancy-related)** assesses changes experienced by the participant to pregnancy and parenting education classes (e.g., "my classes changed to virtual/online"), pregnancy care (e.g., "My ultrasound was cancelled"), birth plans (e.g., "I had to choose between a doula or my partner present for labour and birth"). Scoring was weighted to reflect degree of disruption to find a maximum total score of 35.
Scope50	Assesses the duration and intensity of the COVID-19 crisis at the time of survey completion, based on the participant's state of residence. This score was derived using national and state data available online to calculate two subscales: Duration25 calculated from the number of days since their country, state/territory declared its state of emergency or its first COVID-19 death; and Intensity25, calculated from the total cumulative number of deaths in the country and in the state/territory, and the national and state/territory deaths in the week preceding their recruitment.
SocialDistancing40	Assesses the level of social distancing experienced by the respondent, as measured by reduced in-person contact, and cancelled or avoidance of activities. Comprised of 10 items, weighted for degree of disruption to pre-pandemic life, to find a maximum possible score of 40. For example, "*Reduced in-person contact with family members who live outside the home*".
